# A map of human microRNA variation uncovers unexpectedly high levels of variability

**DOI:** 10.1186/gm363

**Published:** 2012-08-24

**Authors:** José Carbonell, Eva Alloza, Pablo Arce, Salud Borrego, Javier Santoyo, Macarena Ruiz-Ferrer, Ignacio Medina, Jorge Jiménez-Almazán, Cristina Méndez-Vidal, María González-del Pozo, Alicia Vela, Shomi S Bhattacharya, Guillermo Antiñolo, Joaquín Dopazo

**Affiliations:** 1 Institute of Computational Genomics, Centro de Investigación Príncipe Felipe (CIPF), C/ Eduardo Primo Yufera 3, Valencia, 46012, Spain; 2 Medical Genome Project, Andalusian Center for Human Genomic Sequencing, c/ Albert Einstein s/n. Plta. Baja, Sevilla, 41092, Spain; 3 Unidad de Gestión Clínica de Genética, Reproducción y Medicina Fetal. Instituto de Biomedicina de Sevilla (IBIS), Hospital Universitario Virgen del Rocío/CSIC/ Universidad de Sevilla, Avda. Manuel Siurot s/n, Sevilla, 41013, Spain; 4 Centro de Investigación Biomédica en Red de Enfermedades Raras (CIBERER), Avda. Manuel Siurot s/n, Sevilla, 41013, Spain; 5 BIER, Centro de Investigación Biomédica en Red de Enfermedades Raras (CIBERER), CIPF, C/ Eduardo Primo Yufera 3, Valencia, 46012, Spain; 6 Andalusian Molecular Biology and Regenerative Medicine Centre (CABIMER), Avda. Americo Vespucio s/n, Sevilla, 41092, Spain; 7 Functional Genomics Node (INB), CIPF, C/ Eduardo Primo Yufera 3, Valencia, 46012, Spain

## Abstract

**Background:**

MicroRNAs (miRNAs) are key components of the gene regulatory network in many species. During the past few years, these regulatory elements have been shown to be involved in an increasing number and range of diseases. Consequently, the compilation of a comprehensive map of natural variability in a healthy population seems an obvious requirement for future research on miRNA-related pathologies.

**Methods:**

Data on 14 populations from the 1000 Genomes Project were analyzed, along with new data extracted from 60 exomes of healthy individuals from a population from southern Spain, sequenced in the context of the Medical Genome Project, to derive an accurate map of miRNA variability.

**Results:**

Despite the common belief that miRNAs are highly conserved elements, analysis of the sequences of the 1,152 individuals indicated that the observed level of variability is double what was expected. A total of 527 variants were found. Among these, 45 variants affected the recognition region of the corresponding miRNA and were found in 43 different miRNAs, 26 of which are known to be involved in 57 diseases. Different parts of the mature structure of the miRNA were affected to different degrees by variants, which suggests the existence of a selective pressure related to the relative functional impact of the change. Moreover, 41 variants showed a significant deviation from the Hardy-Weinberg equilibrium, which supports the existence of a selective process against some alleles. The average number of variants per individual in miRNAs was 28.

**Conclusions:**

Despite an expectation that miRNAs would be highly conserved genomic elements, our study reports a level of variability comparable to that observed for coding genes.

## Background

Among the non-coding RNA elements, microRNAs (miRNAs) constitute a class of relevant functional and regulatory factors. miRNAs are short non-coding RNAs approximately 22 nucleotides long that play an important role as post-transcriptional regulators [1]. These *trans*-acting factors regulate mRNA functionality by modulating both mRNA stability and the translation of mRNA into protein [2,3]. Some estimates suggest that a relatively large amount of the genes in the human genome (which might comprise up to the 4%) could effectively code for miRNAs. Such regulatory elements present a broad range of targets: it is believed that a single miRNA can regulate the expression of as many as 200 genes [4]. Numerous studies have involved miRNAs in a large number of key biological processes, such as cell growth, proliferation, differentiation, development, and so on [5-7]. As an obvious consequence of this, miRNA deregulation has been associated with a large number of diseases, including cancers, psychiatric and neurological diseases, and so on [8]. As in other functionally relevant elements, and especially due to their crucial regulatory role based on base complementarity, few mutations are expected to occur in miRNAs. Actually, most mutations of the miRNA sequence are expected to have adverse effects on their functionality or biogenesis [9]. Early studies on variability of miRNAs using SNPs reported a very low level of variation in their functional regions [10]. The absence of polymorphisms in more than 90% of human pre-miRNAs has been reported. Moreover, most of the observed polymorphisms occurred outside the seed region, suggesting the existence of a strong selective constraint on human pre-miRNAs [10]. Even common variants located in miRNAs seem to have effects on susceptibility to diseases such as cancer [11,12], ulcerative colitis [13] and many others [8]. SNP genotype data were also used to study the variability at miRNA binding sites. Different authors have also reported a significantly low variation at these sites, thus providing independent support for the notion of high conservation of the miRNA regulatory network [10,14].

The recent publication of the 1000 Genomes Project has revealed an enormous amount of variation at the genome level [15]. An interesting aspect that can be studied from a population perspective is the occurrence of variations across the different genomic features. In particular, variants predicted to severely affect the function of human protein-coding genes, known as loss-of-function (LOF) variants, have attracted the attention of many researchers [15]. Such variants are thought to have a potential deleterious effect and have traditionally been associated with severe Mendelian diseases. However, recent genome sequencing projects have revealed an unexpectedly large number of these variants in the genomes of apparently healthy individuals. A conservative estimate suggests that there are at least 250 LOF variants per sequenced genome, 100 of them involved in known human diseases, and more than 30 in a homozygous state, which suggests a previously unnoticed level of variation with putative functional consequences in protein coding regions in humans [16].

Therefore, it seems urgent to produce a comprehensive catalogue of the natural variation of miRNAs in order to discriminate between pathogenic and non-pathogenic variants in further studies. It is important to check whether a restrictive scenario for the existence of variation is confirmed or, on the contrary, a scenario of variation similar to that observed with coding genes also occurs in miRNAs. In order to distinguish between these two possibilities, we have analyzed the miRNA sequences of 1,152 individuals to survey the actual levels of variability at these genomic elements. The samples belong to 14 populations distributed worldwide, completed with sequencing data corresponding to 60 exomes from the Medical Genome Project [17]. Our analysis discovered a significant number of variants, 527, affecting not only the pre-processed miRNA but also the mature miRNAs. Still, a considerable number of these variants (over 35%) have not yet been reported in dbSNP [18]. The variants found affect different miRNAs known to be involved in 57 diseases. Actually, 41 variants showed a significant deviation from the Hardy-Weinberg equilibrium (HWE), suggesting that selective pressure might be acting against them.

## Materials and methods

### miRNA data sources

**Figure 1:**
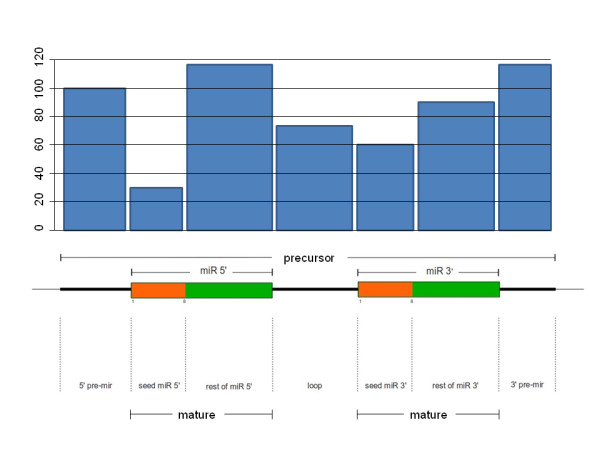
**Structure of a pre-miRNA, an miRNA precursor**. Top: distribution of all the variants observed in all the populations analyzed across the different substructures of the pre-miRNA. Bottom: the theoretical pre-miRNA. The mature miRNA is composed of the seed miR 5' and the rest of miR 5' (and the other mature miRNA is the same for the 3').

miRNAs and their chromosomal coordinates were obtained from miRBase [4] release 18. The regions within miRNAs were defined as in Figure 1 (bottom). The seed region comprising nucleotides 2 to 8 in Figure 1 is a well-known feature of miRNAs [19,20], although recent work extends the region to nucleotide 1 [21]. The definition of the miRNA duplex, including the rest of the elements (pre-mir and the rest of the miR 5' and 3' as well as the loop) was also taken from the literature [22,23]. The associations of miRNAs to diseases were taken from the PhenomiR database [24].

### Human populations

Fourteen human populations were used in this study: the European populations TSI (from Toscani in Italia; 98 samples), FIN (Finnish from Finland; 93 samples), GBR (British from England and Scotland; 89 samples), CEU (Utah residents (CEPH collection) with Northern and Western European ancestry; 85 samples) and IBS (Iberian populations in Spain; 14 samples); the Asian populations CHB (Han Chinese in Beijing, China; 97 samples), CHS (Han Chinese South; 100 samples) and JPT (Japanese in Toyko, Japan; 89 samples); the American populations MXL (Mexican Ancestry in Los Angeles, CA; 66 samples), PUR (Puerto Rican in Puerto Rico; 55 samples) and CLM (Colombian in Medellin, Colombia; 60 samples); and the African populations YRI (Yoruba in Ibadan, Nigeria; 88 samples), LWK (Luhya in Webuye, Kenya; 97 samples) and ASW (African Ancestry in Southwest USA; 61 samples). This selection has been completed with AND, an Andalusian (south of Spain) population composed of 60 samples from healthy individuals, sequenced in the context of the Medical Genome Project [17], which can be considered as equivalent to IBS. AND data have been deposited at the European Genome-phenome Archive [25], which is hosted by the European Bioinformatics Institute, under accession number EGAS00001000324. All these populations together comprise a total of 1,152 individuals.

### Human subjects

The whole genome sequences of the human populations described above (except AND) were downloaded from the 1000 Genomes Project web page [26] in multi-sample VCF format (October 2011 release). Variants in positions located in the miRNAs were collected for this study.

Following informed consent, the 60 AND samples were obtained and further anonymized and sequenced. Samples were obtained in accordance with the approved protocols of the respective institutional review boards for the protection of human subjects. The study conformed to the tenets of the declaration of Helsinki.

### Construction of DNA libraries and sequencing

Library preparation and Exome capture were performed according to protocol version 2.1 from Baylor College of Medicine [27]. Briefly, 5 μg of input genomic DNA was sheared, end-repaired and ligated with SOLID-specific adaptors. A fragment size distribution ranging from 160 to 180 bp after shearing and 200 to 250 bp after adaptor ligation was verified by Bioanalyzer (Agilent, Santa Clara, California, USA). The library was amplified by pre-capture LM-PCR (linker mediated-PCR) using the FastStart High Fidelity PCR System (Roche, Basel, Switzerland). After purification, 2 μg of the LM-PCR product was hybridized to NimbleGen SeqCap EZ Exome libraries. After washing, amplification was performed by post-capture LM-PCR using the FastStart High Fidelity PCR System (Roche). Capture enrichment was measured by quantitative PCR according to the NimbleGen protocol.

The successfully captured DNA was measured by Quant-iT™ PicoGreen^® ^dsDNA reagent (Invitrogen, Carlsbad, California, USA) and subjected to standard sample preparation procedures for sequencing with the SOLID 4 platform as recommended by the manufacturer. Briefly, emulsion PCR was performed on the E20 scale (about 250 million template beads) using a concentration of 0.343 pM of enriched captured DNA. After breaking and enrichment, about 128 million enriched template beads were sequenced per well on a four-well SOLID slide.

### Analysis of sequencing data

The analysis was done using the pipeline of the Medical Genome Project. Briefly, sequence reads were aligned to the reference human genome build GRCh37 (hg19) by using the Burrows-Wheeler alignment (BFAST tool) [28]. Properly mapped reads were filtered with SAMtools [29], which was also used for sorting and indexing mapping files. Only high quality sequence reads with unique mapping positions to the reference human genome were used for calling variants. Identification of variants in the alignment files was performed using the Genome Analysis Toolkit [30]. Known SNPs were obtained from the National Center for Biotechnology Information dbSNP build 135 [31] and the 1000 Genomes Project [26] (October 2011 release).

Characterization of variants across samples was performed by custom applications developed in Java. Each variant profile summarized zygosity, mean coverage, and mean alternative allele frequency across samples. The miRNA identifier and corresponding pre-mir substructure for each variant were obtained by matching variant location with pre-mir coordinates retrieved from miRBase [32].

miRNA variant analysis was performed by custom R [33] scripts. Computed features from analysis were: single nucleotide variant (SNV) frequency (per pre-mir and mature miRNA, per pre-mir substructure, and per subject), subject frequency (per variant and per pre-mir and mature miRNA), disease frequency (per pre-mir), and pre-mir frequency (per subject and disease).

Deviations from the Hardy-Weinberg proportions were tested with a χ^2 ^test [34] as evidence for possible selection against the alternative allele homozygote. Principal component analysis was carried out using the Eigenstrat program [35]. Hive plots were obtained using the R package HiveR [36]. All clustering charts were created using the Heatmap command of R stats package [33].

### Validation of selected variants in miRNA genes by PCR-based direct genomic sequencing

Peripheral blood samples were collected from all subjects for genomic DNA purification using an automated DNA extractor (MagNA Pure LC Instrument, Roche Diagnostics, Basel, Switzerland). In order to validate the variations detected by next-generation sequencing, specific primers were designed using the Primer 3 Output program [37]. PCR conditions and primer sequences employed are available upon request. The amplified products were subsequently purified using an enzymatic procedure, according to the manufacturer's recommendations (EXOSAP-IT^®^, USB Corporation, Cleveland, Ohio, USA) and sequenced with a ready reaction kit (BigDye Terminator Cycle FS Ready Reaction kit; PE-Applied Biosystems, Foster City, CA, USA). The fragments obtained were purified using fine columns (Sephadex G-501, Sigma-Aldrich Co., St. Louis, Missouri, USA) and resolved on an automated sequencer (3730 DNA Analyzer, Applied Biosystems, Carlsbad, California, USA). Finally, the data were analyzed using Lasergene DNASTAR^® ^software (DNASTAR, Inc., Madison, WI, USA).

## Results and discussion

### Variants found in miRNAs

The capture kit used in the exome enrichment (see Materials and methods) contains 720 miRNAs. In this study we have focused on these regions, as representative of the variability of the whole spectrum of human miRNAs. In the case of the 14 populations from the 1000 Genomes Project, the genomic variant files (VCF) were downloaded from the 1000 Genomes Project web site and mapped over the genomic positions of the 720 miRNAs. The variants corresponding to these regulatory elements were considered for the study. In the case of the newly sequenced Spanish samples, the sequencing reads were mapped onto the chromosomal coordinates of miRNAs and the variants were called as described in Materials and methods. The average coverage observed in these regions was satisfactory (approximately 40×) and the frequency of the alternative allele when the variant call was heterozygous was over 30% of the reads. These results ensured the quality of the variant calling process.

A total of 527 different sites located within the complete pre-miRNA structures of the 1,152 individuals analyzed were affected by SNVs. Table 1 summarizes the SNVs found in this study and Additional file 1 contains a comprehensive description of the variability map found in human miRNAs. Thirty-five percent of these SNVs were not present in dbSNP, indicating that massive sequencing still has considerable potential for variant discovery. Also among these SNVs, 45 affected the 5' and 3' seed regions and occurred in 44 different miRNAs. Sixty percent of these miRNAs (26) have previously been linked to diseases, according to the human miRNA disease databases [8,24]. In the case of the 60 new Spanish individuals sequenced, 11 so far undescribed variants were found (Table 2) among the 92 SNVs detected. Almost all of them were unique variants. Two of them could not be validated (see Materials and methods) due to technical problems. Actually, hsa-mir-1324 validation was inconclusive because multiple bands appeared in the gel. When this miRNA was checked in miRBase, we found that it was withdrawn because of poor structure (entry MI0006655) but it was included again (entry MI0006657). This entry contains a note of caution because some additional sequences reported for this miRNA [38] did not meet miRBase requirements for miRNA identification. This could explain the difficulties found in its validation.

**Table 1:** Summary of variant positions found

Origin	Population	Subjects	SNVs	SNVs in dbSNP	SVNs in precursor	SVNs in mature	Number of heterozygous	Number of homozygous	miRNA with disease	Diseases
European	IBS	14	78	75	70	15	52	26	21	76
	AND	60	92	77	81	21	55	37	30	82
	CEU	85	132	119	116	29	87	45	42	102
	TSI	98	147	127	120	32	104	43	120	109
	GBR	89	131	114	110	28	80	51	43	107
	FIN	93	118	103	102	25	72	46	36	95
Asian	CHS	100	114	92	93	28	65	49	30	88
	CHB	97	123	99	104	25	77	46	30	91
	JPT	89	120	108	103	32	72	42	38	96
American	MXL	66	132	111	110	34	82	50	38	90
	PUR	55	152	141	117	35	107	45	41	85
	CLM	60	141	124	114	28	90	51	37	88
African	LWK	97	229	187	179	59	158	71	70	104
	ASW	61	206	177	158	54	145	60	60	108
	YRI	88	207	175	169	53	130	77	169	133

**Figure 2:**
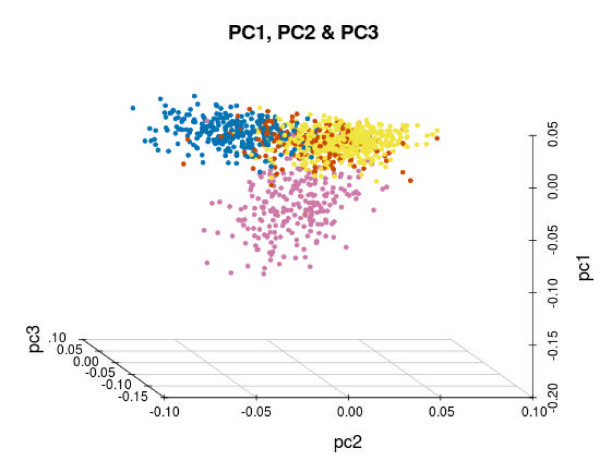
**Principal component analysis based on the variability of the 14 populations from the 1000 Genomes Project and the Spanish sequenced population**. Distances are based on a total of 198 different variant positions in which the sequenced Spanish population presented a reasonable coverage (>10×) and there was at least one variant in an individual. Colors are as follows: European populations in yellow; Asian populations in blue; African populations in purple and American populations in brown. PC, principal component.

**Figure 3:**
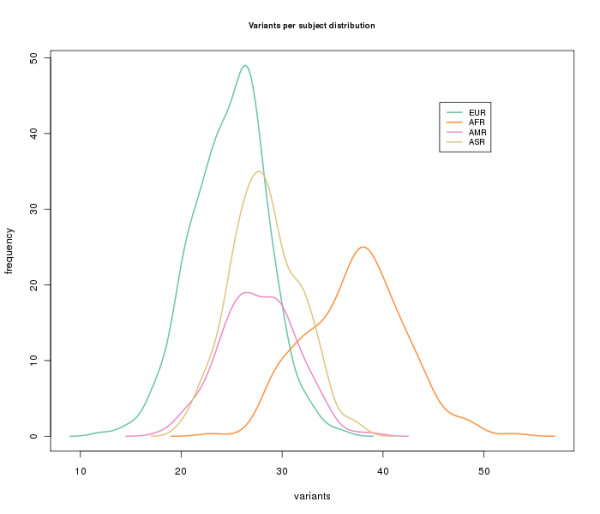
**Distribution of the number of variants per individual in the different geographical origins derived from the populations analyzed**. The populations used for the different geographical origins were: EUR (European), including TSI, FIN, GBR, CEU, IBS and AND; ASI (Asian), including CHB, CHS and JPT); AMR (American) (including MXL, PUR and CLM); and AFR (African), including YRI, LWK and ASW.

Origin, geographical origin of the population; Population, population code according to the 1000 Genomes Project (see Materials and methods); Subjects, number of sequenced subjects per population; SNVs, number of SNVs found in the population; SNVs in dbSNP, how many of these variants are already described in dbSNP; SVNs in precursor, how many of the SNVs map within the miRNA precursor structure (Figure 3, bottom); SVNs in mature, how many of the SNVs map within the miRNA mature structure (Figure 2, bottom); Number of heterozygous, the number of times that the SNV occurred heterozygously; Number of homozygous, the number of times that the alternative allele occurred homozygously; miRNA with disease, the number of miRNAs associated with at least one known disease, with at least one SNV, in the corresponding population; Diseases, the total number of diseases with which the miRNAs are associated.

**Table 2:** New variants found in the newly sequenced Spanish population (AND) arranged by chromosome location

Location	SNV	Pre-mir/mature	Region	Number of individuals	Population frequency (%)	Number of heterozygous	Number of homozygous	Validated
Chr1:236016354	C/G	hsa-mir-1537	5' mir (mix)	1	0.0868	1	0	Yes
Chr 3:10436198	G/A	hsa-mir-885/hsa-miR-885-3p	3' seed miR	1	0.0868	1	0	Yes
Chr 3:75679944	C/T	hsa-mir-1324	5' mir (mix)	2	0.1736	2	0	NC^a^
Chr 4:8007067	G/A	hsa-mir-95	5' mir (mix)	1	0.0868	1	0	Yes
Chr 9:97847780	C/T	hsa-mir-27b	Loop	1	0.0868	1	0	Yes
Chr 12:49048256	G/A	hsa-mir-1291	3' mir (mix)	1	0.0868	1	0	Yes
Chr 14:23887249	C/G	hsa-mir-208b	5' mir (mix)	1	0.0868	1	0	ND^b^
Chr 14:101347358	C/T	hsa-mir-431	5' mir	1	0.0868	1	0	Yes
Chr 14:101506800	G/C	hsa-mir-376b	5' mir (mix)	1	0.0868	1	0	Yes
Chr 14:101513665	A/T	hsa-mir-539	5' mir	1	0.0868	1	0	Yes
Chr 14:101531692	T/C	hsa-mir-409/hsa-miR-409-3p	3' rom miR	1	0.0868	1	0	Yes

Location, the location (chromosome:position, according to the human genome build GRCh37, hg19); SNV, the change observed (reference allele/alternative allele); Pre-mir/mature, the identifier of the pre-mir and the mature miRNA affected by the SNV; Region, the region in which the SNV maps within the miRNA structure (Figure 3, bottom), with several cases where regions cannot be defined with precision due to a lack of a detailed annotation (3' mir (mix) = loop + seed miR 3' + rest of miR 3' + 3' pre-mir; 5' mir (mix) = loop + seed miR 5' + rest of miR 5' + 5' pre-mir; we also use the abbreviations 5' rom miR = rest of miR 5' and 3' rom miR = rest of miR 3'); Number of individuals, the number of individuals supporting the SNV; Population frequency (%), the alternative allele frequency in the studied population; Number of heterozygous, the number of times the SNV occurred heterozygously; Number of homozygous, the number of times that the alternative allele occurred homozygously. ^a^NC, non-conclusive results after using two independent pairs of primers; a double band makes a precise determination of the SNV impossible. ^b^ND, not determined because no more DNA was available.

While previous estimations of variability suggested that only about 10% of the miRNAs will be affected by polymorphisms [10], our study reports 44% have variants in the analyzed populations. Moreover, 17% contain variants in critical regions of the mature miRNA. Another possible comparison comes from the 1000 Genomes Project, which recently reported an unexpected amount of deleterious mutations in the coding genes of approximately 250 to 300 of these LOF variants per individual [15]. Considering 21,160 protein coding genes (according to Ensembl, human genome version GRCh37.p5), there is a ratio of 0.014 LOF variants per gene in the genome. If we consider those SNVs located in the seed regions as potentially functional variants, the equivalent resulting ratio would be 0.05 functional variants per miRNA, which is 3.5-fold higher than for protein coding regions. Despite the simplicity of this comparison, it reveals a scenario with a level of variability higher than previously expected.

The distribution of SNVs in the miRNAs across individuals in different populations is consistent with the known historical divergences among populations [39,40]. Figure 2 shows a principal component analysis that clearly segregates the four main populations: African, European, Asian and American. Human population migrations is a well-known factor in the determination of current patterns of intra- and inter-population genetic diversity [41]. Figure 3 shows the distribution of SNVs per individual for the different geographical origins, derived from the analyzed populations. African populations, as expected, display the highest variability: 37.2 variants per individual mapping onto miRNAs, versus averages ranging between 24 and 29 in the other geographical origins.

The distribution of variants among the different miRNAs is not uniform. While most of them were affected by only one SNV, 19 mature miRNAs are affected by 2 SNVs, among which are hsa-miR-302b*, associated with carcinoma, glioma, embryonal carcinoma and prostatic, thyroid and skin neoplasms, and hsa-miR-629, associated with liver, colonic and testicular neoplasms and lymphomas, according to the Human MicroRNA Disease database [8]. In addition, two mature miRNAs are affected by three SNVs, hsa-miR-323b-5p and hsa-miR-936, with no known association to any disease described to date. This observation indicates that multiple mutations rarely occur in miRNAs associated with diseases.

The variants were not homogeneously distributed across the structure of the pre-miRNA. Figure 1 (top) shows the distribution of the occurrence of variants along the different pre-miRNA substructures. As expected, the regions corresponding to the mature miRNA as well as the loop (see Figure 1 (bottom) for the description of the miRNA regions) displayed a lower occurrence of variants than the rest of the miR 5' and 3' regions. Table 3 contains a comprehensive description of all the SNVs located in the seed regions, putatively critical for the functionality of miRNA, which occurs by complementarity with their respective targets. A total of 45 SNVs were found in these critical regions, which comprise 8% of the total variants found.

**Table 3:** SNVs found in all the populations analysed affecting critical regions of miRNAs

Location	SNV	Pre-mir/mature	[dbSNP]/[1000g aaf]	Region	Number of samples	Population frequency (%)	Number of heterozygous	Number of homozygous
1:17185500	C/T	hsa-mir-3675/hsa-miR-3675-5p	RS137982850/-	5' seed miR	1	0.0868	0	1
1:100746814	A/G	hsa-mir-553/hsa-miR-553	-/0.00	5' seed miR	3	0.2604	3	0
1:176998565	T/C	hsa-miR-488-5p	-/0.00	5' seed miR	1	0.0868	1	0
3:164059159	G/A	hsa-mir-720/hsa-miR-720	-/0.00	5' seed miR	1	0.0868	1	0
4:113569088	A/G	hsa-miR-367-5p	RS150161032/0.00	5' seed miR	1	0.0868	1	0
5:140027435	C/T	hsa-mir-3655/hsa-miR-3655	RS146400503/0.01	5' seed miR	13	1.1285	13	0
7:73605546	C/T	hsa-mir-590/hsa-miR-590-5p	-/0.00	5' seed miR	4	0.3472	4	0
7:127721934	C/T	hsa-miR-593-5p	RS73721294/0.02	5' seed miR	42	3.6458	40	2
10:29891260	C/T	hsa-mir-938/hsa-miR-938	RS12416605/0.15	5' seed miR	301	26.1285	261	40
10:53059349	A/G	hsa-mir-605/hsa-miR-605	RS113212828/0.00	5' seed miR	8	0.6944	8	0
10:105807928	T/C	hsa-mir-936/hsa-miR-936	RS79924817/0.01	5' seed miR	11	0.9549	11	0
13:53384209	C/G	hsa-mir-759/hsa-miR-759	RS144233096/0.00	5' seed miR	4	0.3472	4	0
14:101520657	T/C	hsa-miR-382-5p	-/0.00	5' seed miR	1	0.0868	1	0
15:42491848	A/C	hsa-mir-627/hsa-miR-627	RS2620381/0.08	5' seed miR	157	13.6285	145	12
19:46178206	T/G	hsa-miR-642a-5p	RS78902025/0.00	5' seed miR	1	0.0868	1	0
1:3477292	C/T	hsa-mir-551a/hsa-miR-551a	-/0.00	3' seed miR	10	0.8681	10	0
1:168344829	C/T	hsa-mir-557/hsa-miR-557	RS78825966/0.06	3' seed miR	114	9.8958	98	16
2:176032384	T/C	hsa-mir-933/hsa-miR-933	RS139770589/0.00	3' seed miR	8	0.6944	8	0
3:10436198	G/A^a^	hsa-mir-885/hsa-miR-885-3p	New variant^a^	3' seed miR	1	0.0868	1	0
3:49311593	G/C	hsa-mir-4271/hsa-miR-4271	-/0.00	3' seed miR	2	0.1736	2	0
3:160122434	G/A	hsa-miR-15b-3p	-/0.00	3' seed miR	1	0.0868	1	0
4:1988144	C/A	hsa-mir-943/hsa-miR-943	-/0.00	3' seed miR	2	0.1736	2	0
4:83674520	G/A	hsa-mir-575/hsa-miR-575	RS149186367/0.00	3' seed miR	10	0.8681	10	0
4:117220909	G/A	hsa-mir-1973/hsa-miR-1973	-/0.00	3' seed miR	1	0.0868	1	0
5:54468124	A/T	hsa-miR-449c-3p	RS35770269/0.25	3' seed miR	460	39.9306	368	92
5:148810267	C/T	hsa-miR-145-3p	-/0.00	3' seed miR	1	0.0868	1	0
5:159912418	C/G	hsa-miR-146a-3p	RS2910164/0.62	3' seed miR	933	80.9896	458	475
5:168690635	C/T	hsa-mir-585/hsa-miR-585	RS62376935/0.10	3' seed miR	199	17.2743	176	23
7:150935577	G/A	hsa-mir-671/hsa-miR-671-3p	-/0.00	3' seed miR	1	0.0868	1	0
8:10682898	A/G	hsa-mir-1322/hsa-miR-1322	-/0.00	3' seed miR	5	0.434	5	0
10:29833959	C/T	hsa-mir-604/hsa-miR-604	-/0.00	3' seed miR	3	0.2604	3	0
12:113132901	G/A	hsa-mir-1302-1/hsa-miR-1302	RS74647838/0.03	3' seed miR	53	4.6007	50	3
14:101351088	A/C	hsa-miR-136-3p	RS147448304/0.00	3' seed miR	1	0.0868	1	0
16:820249	G/A	hsa-mir-662/hsa-miR-662	RS9745376/0.05	3' seed miR	97	8.4201	87	10
16:2321809	G/A	hsa-mir-940/hsa-miR-940	RS149527765/0.00	3' seed miR	1	0.0868	1	0
18:56118358	C/T	hsa-miR-122-3p	RS41292412/0.00	3' seed miR	10	0.8681	10	0
19:2234080	G/A	hsa-mir-1227/hsa-miR-1227	RS139405773/0.00	3' seed miR	2	0.1736	2	0
19:10662866	G/A	hsa-mir-1238/hsa-miR-1238	-/0.00	3' seed miR	1	0.0868	1	0
19:10928119	A/G	hsa-mir-199a-1/hsa-miR-199a-3p	-/0.01	3' seed miR	13	1.1285	13	0
19:54200843	C/T	hsa-mir-525/hsa-miR-525-3p	-/0.00	3' seed miR	1	0.0868	1	0
19:54201692	A/C	hsa-miR-523-3p	-/0.00	3' seed miR	3	0.2604	3	0
19:54238189	G/A	hsa-mir-518d/hsa-miR-518d-3p	RS73602910/0.00	3' seed miR	6	0.5208	6	0
20:33578251	A/G	hsa-miR-499a-3p	RS3746444/0.18	3' seed miR	358	31.0764	311	47
20:33578255	C/T	hsa-miR-499a-3p	RS150018420/0.00	3' seed miR	3	0.2604	3	0
22:42296995	A/G	hsa-miR-33a-3p	RS77809319/0.00	3' seed miR	2	0.1736	2	0

Location, the location (chromosome:position, according to the human genome build GRCh37, hg19); SNV, the change observed (reference allele/alternative allele); Pre-mir/mature, the identifier of the pre-mir and the mature miRNA affected by the SNV; [dbSNP]/[1000g aaf], the dbSNP identifier and the 1000 Genomes alternative allele frequency; Region, the region in which the SNV maps within the miRNA structure (Figure 1, bottom); Number of samples, the number of samples supporting the SNV; Population frequency (%), the alternative allele frequency in the studied population; Number of heterozygous, the number of times that the SNV occurred heterozygously; Number of homozygous, the number of times that the alternative allele occurred homozygously. ^a^This new SNV found in hsa-mir-885/hsa-miR-885-3p was experimentally confirmed (Table 2).

### Zygosity, allele frequencies and selective pressures

**Figure 4:**
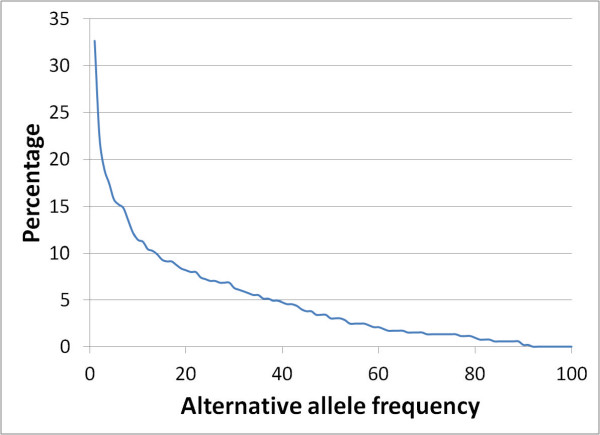
**Number of variant positions with different allelic frequencies**. Distribution of SNVs according to their relative allelic frequencies.

As expected, most of the variants found in the studied samples were heterozygotes (399 out of 527, approximately 75%). However, for one-fourth of the variants detected (128) the alternative allele was homozygous in at least one individual. Also according to the expectations, allele population frequencies were low for most SNVs. Figure 4 shows the distribution of variants according to their allele frequency estimated from the populations used in this study.

A classical way to verify the existence of variants under negative selection (and, consequently, probably pathogenicity) is to check for deviations from HWE [34]. HWE states that, given a series of assumptions, both allele and genotype frequencies in a population remain constant (in equilibrium) with time unless specific disturbing influences are introduced. Deviations from this equilibrium can be tested with a conventional chi-square test. Selective pressures against one of the alleles are one of the most common factors that can produce this deviation. Table 4 shows 41 SNVs belonging to all the populations analyzed that were significantly deviated from the HWE. Actually, the real number might be higher because the small sizes of the populations available for the study (around or below 100 individuals) preclude the possibility of obtaining lower *P*-values even for highly deviated proportions.

**Table 4:** Variant positions with significant deviations from Hardy-Weinberg equilibrium

Population	Location Chr:position	SNV	Pre-mir/mature	[dbSNP]/[1000g aaf]	Region	Number of subjects	Number of heterozygous	Number of homozygous	HWE *P*-value
AND	5:159912418	C/G	hsa-miR-146a-3p	RS2910164/0.62	3' seed miR	35	13	22	2.82 × 10^-05^
	10:135061112	C/T	hsa-mir-202	RS12355840/0.71	5' mir	28	2	26	2.09 × 10^-12^
	X:49767835	A/G	hsa-mir-532	RS456617/-	3' mir	14	4	10	6.87 × 10^-09^
ASW	2:241395503	T/C	hsa-mir-149	RS2292832/0.58	3' mir	58	35	23	0.044409715
	4:1988193	T/C	hsa-mir-943	RS1077020/0.26	5' mir (mix)	32	15	17	0.000302955
	14:101522589	C/T	hsa-mir-323b/hsa-miR-323b-5p	RS75330474/0.03	5' rom miR	3	2	1	0.00016146
	15:70371772	C/T	hsa-miR-629-5p	RS111899904/0.01	5' rom miR	3	2	1	0.00016146
	20:2633466	C/T	hsa-mir-1292	RS73576045/0.01	3' mir (mix)	3	2	1	0.00016146
CEU	4:115577997	C/G	hsa-mir-577	RS34115976/0.12	3' mir (mix)	22	17	5	0.048957519
	7:129414574	A/G	hsa-mir-96	RS41274239/0	Loop	1	0	1	<10^-15^
	17:28444183	A/C	hsa-mir-423	RS6505162/0.51	3' mir	64	57	7	0.000893732
	22:23165340	C/G	hsa-mir-650	RS5996397/0.15	3' mir (mix)	22	15	7	0.001677244
CHB	8:145625538	C/G	hsa-mir-1234	RS141140965/0.38	5' mir (mix)	83	66	17	0.000639166
	17:28444183	A/C	hsa-mir-423	RS6505162/0.51	3' mir	91	20	71	0.030681656
	20:58883605	T/G	hsa-mir-646/hsa-miR-646	RS6513497/0.19	3' rom miR	36	36	0	0.049947315
CHS	7:129410227	C/T	hsa-mir-182	RS76481776/0.05	3' mir	3	2	1	9.68 × 10^-07^
	8:145625538	C/G	hsa-mir-1234	RS141140965/0.38	5' mir (mix)	84	71	13	4.85 × 10^-05^
CLM	1:19223639	G/A	hsa-mir-1290	RS75705742/0.01	5' mir (mix)	1	0	1	9.44 × 10^-15^
	2:180725568	T/C	hsa-mir-1258	RS146754630/0.01	3' mir	2	1	1	3.44 × 10^-07^
	5:36148057	T/C	hsa-mir-580	RS115089112/0.01	5' mir (mix)	2	1	1	3.44 × 10^-07^
FIN	8:113655752	T/C	hsa-mir-2053	RS10505168/0.37	5' mir (mix)	57	51	6	0.047377863
	18:33484792	G/A	hsa-mir-187	RS41274312/0.01	3' mir	3	2	1	2.41 × 10^-06^
GBR	12:95228286	G/C	hsa-mir-492	RS2289030/0.12	3' mir (mix)	8	6	2	0.01260062
	14:100774203	C/T	hsa-mir-345	RS72631832/0.01	5' mir	7	5	2	0.004139358
IBS	20:58883534	T/C	hsa-mir-646	RS6513496/0.24	5' mir (mix)	3	1	2	0.039045114
	22:23165340	C/G	hsa-mir-650	RS5996397/0.15	3' mir (mix)	3	1	2	0.039045114
JPT	2:47604866	C/T	hsa-mir-559	RS58450758/0.2	3' mir (mix)	31	23	8	0.039987277
	10:53059406	T/C	hsa-mir-605	RS2043556/0.28	3' mir (mix)	39	27	12	0.025845926
	19:54240174	G/A	hsa-miR-516b-3p	RS78861479/0.02	3' rom miR	13	8	5	2.23 × 10^-05^
LWK	8:145625538	C/G	hsa-mir-1234	RS141140965/0.38	5' mir (mix)	59	53	6	0.040183314
	14:101522556	T/C	hsa-mir-323b	RS56103835/0.3	5' mir	3	2	1	1.43 × 10^-06^
	20:62551199	T/C	hsa-mir-941-4;hsa-mir-941-3	RS7360929/0.09	5' mir (mix)	15	12	3	0.039190789
	20:62574006	A/G	hsa-mir-647	RS73147065/0.23	3' mir (mix)	64	36	28	0.017952613
MXL	12:104324266	G/A	hsa-mir-3652	RS17797090/0.06	3' mir (mix)	3	2	1	8.32 × 10^-05^
PUR	2:241395500	A/G	hsa-mir-149	RS71428439/0.14	3' mir	19	13	6	0.034768421
	7:158325503	C/T	hsa-mir-595	RS4909237/0.18	5' mir (mix)	19	12	7	0.008555965
	10:14478618	T/C	hsa-mir-1265	RS11259096/0.09	3' mir (mix)	3	1	2	1.18 × 10^-05^
TSI	5:179225324	G/A	hsa-mir-1229	RS2291418/0.03	5' mir (mix)	4	2	2	1.25 × 10^-06^
	8:145625538	C/G	hsa-mir-1234	RS141140965/0.38	5' mir (mix)	54	51	3	0.016552142
YRI	2:47604866	C/T	hsa-mir-559	RS58450758/0.2	3' mir (mix)	60	54	6	0.006410575
	8:145625538	C/G	hsa-mir-1234	RS141140965/0.38	5' mir (mix)	52	48	4	0.026373978

Population, the population where the SNV was found; Location Chr:position, the location (chromosome:position, according to the human genome build GRCh37, hg19) of the SNV; SNV, the change observed (reference allele/alternative allele); Pre-mir/mature, the identifier of the pre-mir and the mature miRNA when the SNV affects it; [dbSNP]/[1000g aaf], identifiers in dbSNP and/or 1000 Genomes when already described; Region, the region in which the SNV maps within the miRNA structure (Figure 1, bottom); Number of subjects, the number of individuals supporting the SNV; Number of heterozygous, the number of times that the SNV occurred heterozygously; Number of homozygous, the number of times that the alternative allele occurred homozygously; HWE *P*-value, the *P*-value of the test for Hardy-Weinberg deviation, obtained considering the corresponding population.

Again, comparisons to the number of LOF variants in coding genes can be done if SNVs significantly deviated from HWE are considered as potentially functional. In this case, the resulting ratio would be 0.08 functional variants per variant miRNA. This value is 5.5-fold higher than the ratio of LOF variants observed in protein coding regions.

### Diseases associated with miRNAs with variants and potential consequences

The process of human migrations in the past has played a key role in determining current patterns of genetic diversity and could partially explain inter-population variations in genetic susceptibility to certain diseases [41,42]. As previously discussed, variants affecting essential regions of the miRNAs, such as the seed or the loop, have a high probability of having consequences on miRNA functionality. Moreover, it has been described that variants affecting other regions of the miRNA structure can also adversely effect its functionality [9]. Given that miRNAs target many different mRNA sequences, SNVs affecting them are expected to cause considerable pleiotropic effects. Moreover, given that the mode of action of miRNAs implies interference of the mRNAs of their target genes with consequent inhibition of the corresponding gene product, a dominant or codominant (having incomplete penetrance) effect would be expected from a variant that affects an miRNA's functionality. Additional file 2 lists the diseases in which different miRNAs with variants in the studied population have been reported to be involved, according the PhenomiR human miRNA disease database [24].

**Figure 5:**
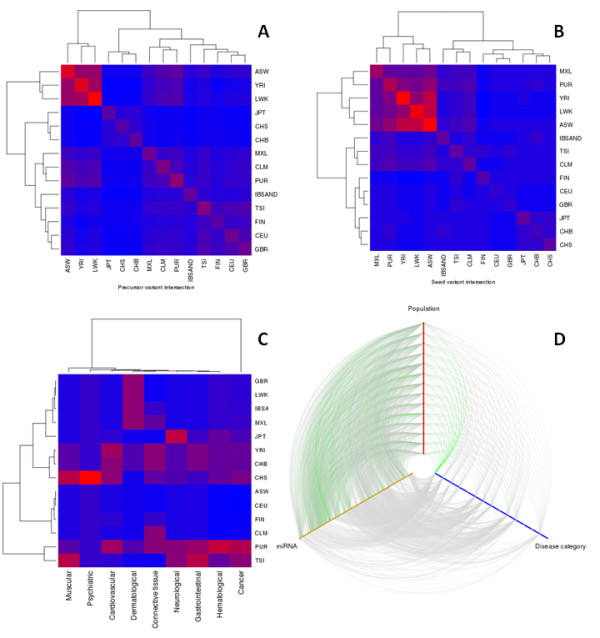
**Relationships between miRNAs, diseases in which they are involved and populations**. Heat maps were produced using an agglomerative clustering algorithm with Euclidean distances. **(a) **Heat map portraying the similarity between populations in terms of shared variants at each position of the miRNA precursor compared. The color code represents the number of common variants between population pairs. **(b) **Heat map of similarity between populations when only those positions mapping in the seed regions of the miRNAs are considered in the comparison. The color code represents the number of common variants between population pairs with the analysis restricted to variants mapping in the seed regions. **(c) **Heat map of the distribution of diseases across populations. The color code represents the number of variants per population associated with each disease category. **(d) **Hive plot showing the relationships between miRNAs, populations and diseases, with the diseases summarized in general categories.

Among the 527 variants found in this work, 45 affected the recognition region of the corresponding miRNA and were found in 43 different miRNAs, 26 of which are known to be involved in 57 diseases. Many of these diseases are cancers, probably because the studies are quite recent and have been carried out mainly from this perspective. However, many miRNAs are involved in other diseases of different etiologies; the fact that a considerable number of them have been associated with more than one disease highlights the pleiotropic effect of miRNAs. This pleitropic effect makes it difficult to understand the relationships between mutations and the possible effects in cases of miRNAs associated with diseases. Figure 5a shows the similarity between populations in terms of shared variants at each position of the miRNA precursor compared. Obviously, intra-population similarities are higher than inter-population similarities. Populations from a common geographical region display higher similarities than when they are compared to populations of other geographical origins. Overall, the resulting clustering obtained for the populations reflects their historic origins, as expected [41]. However, when only positions mapping in the seed regions of the miRNA are considered in the comparison, several populations share more common SNVs with other populations of different geographical origins (Figure 5b) than with neighbor populations. For example, some African, European and American populations seem to be more similar to each other than to other populations with closer geographic origins. This can be explained by the limited repertoire of mutations that can be tolerated in regions critical for miRNA functionality. This population intermingling effect is still more noticeable when the distribution of diseases across populations is visualized (Figure 5c). In this case, the effect is a combination of two factors: on one hand the limited number of miRNAs affected in the critical regions and, on the other, the pleiotropic effect of such miRNAs, which causes a non-proportional distribution of the diseases. As expected, Africans, due to their higher variability levels, cover a wider range of potential diseases. However, the number of diseases is not directly proportional to the general variability levels of the population and depends on the particular repertoire of miRNAs affected. The interrelationships between diseases, populations and miRNAs can be represented by means of a hive plot. Figure 5d shows such relationships, with the diseases summarized in general categories. A complex correspondence between miRNAs and the respective diseases caused is evident. Almost all the miRNAs appear mutated in almost all the populations and the same can be said for the diseases. However, Figure 5a shows some population trends in the diseases, probably due to founder effects.

### Putative functional impact

**Figure 6:**
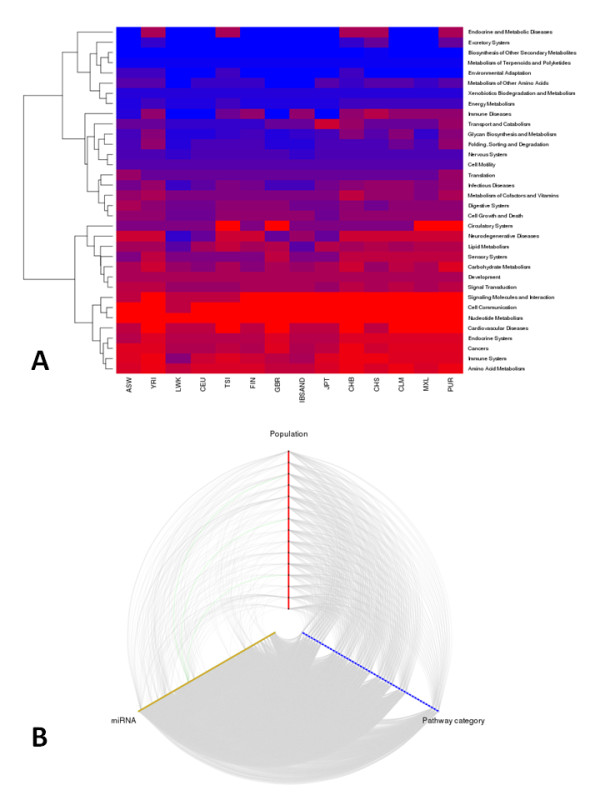
**Relationships between miRNAs, pathway categories and populations**. **(a) **Heat map of the distribution of pathway categories across populations. Color code represents the percentage of pathways potentially altered within any of the categories. A pathway is considered to be affected if one of its genes is targeted by any miRNA with variants in the seed region. **(d) **Hive plot showing the relationships between miRNAs, populations and pathway categories.

Defective miRNAs cause diseases because of their inability to silence the corresponding natural set of target genes (and eventually by silencing new target genes). Since miRNAs have multiple targets, it is expected that they have a collective action over cell functionalities. Indeed, cases of miRNAs acting on genes involved in particular functional processes have been described [43-45]. In order to understand the possible impact of the corresponding population mutational load, we have listed the genes targeted by the 45 different miRNAs with SNVs in their seed regions and selected the KEGG pathways [46] containing genes on this list. A total of 34 (of a total of 51) KEGG pathway categories contained genes targeted by the 45 miRNAs with seed regions affected by SNVs. Figure 6a shows the distribution of putatively affected pathways across populations. Despite the large number of targets affected by the miRNAs, 17 categories are untargeted by any miRNA, all of which are related to basic metabolism, replication, transcription, repair and other basic cellular functions. Actually, several metabolism categories (for example, energy metabolism, metabolism of terpeniods and polyketides, biosynthesis of other secondary metabolites) are among the less affected categories across all the populations. On the other side of the spectrum, pathway categories related to cancer, immune system, cardiovascular diseases and diverse forms of signaling are among the most affected ones. This observation is in accordance with the diseases to which the miRNAs affected have been linked. Again, the patterns of distribution of diseases for African populations are less homogeneous than those of other populations. For example, in LWK populations pathways of neurodegenerative diseases and immune system seem to be less affected than in the other two African populations (YRI and ASW). There are also some interesting distributions of patterns. For example, for some populations of different origins (LKW, GBR and JPT), pathways related to neurodegenerative diseases seem to be less affected than other categories. As in the case of diseases, the relationships among miRNAs, pathway categories and populations are complex, as represented in Figure 6b.

## Conclusions

An increasing amount of evidence accumulated during the past few years firmly links miRNAs to numerous diseases [8,47-49]. Moreover, non-coding RNAs have been proposed as therapeutic agents given their silencing capabilities [50]. Therefore, detailed knowledge on natural variation in miRNAs is needed to discriminate pathological variants from those present in healthy populations [51,52].

The use of a reasonably large sample of 1,152 individuals from 15 different populations from 4 geographical origins allowed us to compile a quite comprehensive catalogue of natural variation in miRNAs. This regulatory element is expected to be under strong negative selection and, consequently, highly conserved [10]. However, our results document an unexpected amount of variability, comparable (actually higher) to what was recently described for coding regions [15].

Although it is difficult to properly judge the real role of this variability, some observations would suggest that, most likely, a functional impact cannot be ruled out. Firstly, the occurrence of SNVs across the complete pre-miRNA structure (Figure 1) is consistent with the relative importance of the distinct miRNA regions to functionality. Obviously, the seed sequence is crucial for miRNA functionality, but other regions, such as those next to the seed, have also recently been involved in miRNA functionality [9]. In the seed regions alone, 45 SNVs disrupt the recognition site of the miRNA for its targets. In addition, as expected, heterozygotic variants are more abundant than the alternative allele homozygotic variants (75.7% versus 24.3%). Moreover, many of the variants found are low-frequency, rare variants or even variants specific to individuals. Obviously, approaches based on rare variants to infer functional impact [53] consider low frequency as a necessary but not sufficient condition to justify the existence of a selective pressure against such variants. On the other hand, selective pressures can also be inferred by deviations from HWE [34], which occurs for 41 of these variants.

Although it is impossible to discard the possibility that any of the healthy controls used in this study will develop some pathology in the future, it is unlikely that this occurs for a significant number of them. Therefore, the apparent neutral effect of potentially deleterious SNVs found in this study might be either a consequence of a recessive effect in functionality or due to the fact that they are neutral changes (despite the fact that they disrupt regions apparently essential for miRNA functionality).

It is likely that that many of the variants discovered that affect essential miRNA regions do have a recessive effect. Then, the probability of generation of homozygosis by random crossing is non-negligible given the allelic frequencies. This fact would also suggest that the role of miRNAs in disease could be stronger than previously suspected. In any case, the role of such recently discovered variability in shaping the phenotype or in the occurrence of diseases is still unclear and deserves further study.

## Abbreviations

bp: base pair; HWE: Hardy-Weinberg equilibrium; LOF: loss of function; miRNA: microRNA; PCR: polymerase chain reaction; SNP: single nucleotide polymorphism; SNV: single nucleotide variant.

## Competing interests

The authors declare that they have no competing interests

## Authors' contributions

JC performed the sequence analysis, helped by EA, PA, JS and IM; SB and MR selected and phenotyped the samples used. The sequencing of the Spanish samples was carried out by AV. The work was coordinated by JD, SSB and GA. JD conceived the work and wrote the manuscript with GA. All authors have read and approved the manuscript for publication.

## Supplementary Material

Additional file 1 **Variant positions found**. Excel file containing a comprehensive list of the 527 variant positions found in the study. Click here for file

Additional file 2 **Diseases in which miRNAs with variant positions have been involved**. Excel file containing a comprehensive list of diseases in which miRNAs with their seed regions affected by SNVs have been involved, according to the human microRNA disease database. Click here for file
